# Fast-track surgery improves postoperative clinical recovery and cellular and humoral immunity after esophagectomy for esophageal cancer

**DOI:** 10.1186/s12885-016-2506-8

**Published:** 2016-07-11

**Authors:** Lantao Chen, Lixin Sun, Yaoguo Lang, Jun Wu, Lei Yao, Jinfeng Ning, Jinfeng Zhang, Shidong Xu

**Affiliations:** Department of Thoracic Surgery, Harbin Medical University Cancer Hospital, Harbin, Heilongjiang Province China; Department of Thoracic Surgery, The Fourth Affiliated Hospital of Harbin Medical University, Harbin, Heilongjiang Province China; Department of Thoracic Surgery, Hainan Cancer Hospital, Haikou, Hainan Province China

**Keywords:** Esophageal cancer, Fast-track surgery, Cellular immunity, Humoral immunity

## Abstract

**Background:**

Our aim was to investigate the influence of FTS on human cellular and humoral immunity using a randomized controlled clinical study in esophageal cancer patients.

**Methods:**

Between October 2013 and December 2014, 276 patients with esophageal cancer in our department were enrolled in the study. The patients were randomized into two groups: FTS pathway group and conventional pathway group. The postoperative hospital stay, hospitalization expenditure, and postoperative complications were recorded. The markers of inflammatory and immune function were measured before operation as well as on the 1st, 3rd, and 7th postoperative days (POD), including serum level of interleukin-6 (IL-6), C-reactive protein (CRP), serum globulin, immunoglobulin G (IgG), immunoglobulin M (IgM), immunoglobulin A (IgA) and lymphocyte subpopulations (CD3 lymphocytes, CD4 lymphocytes, CD8 lymphocytes and the CD4/CD8 ratio) in the patients between the two groups.

**Results:**

In all, 260 patients completed the study: 128 in the FTS group and 132 in the conventional group. We found implementation of FTS pathway decreases postoperative length of stay and hospital charges (*P* < 0.05). In addition, inflammatory reactions, based on IL-6 and CRP levels, were less intense following FTS pathway compared to conventional pathway on POD1 and POD3 (*P* < 0.05). On POD1 and POD3, the levels of IgG, IgA, CD3 lymphocytes, CD4 lymphocytes and the CD4/CD8 ratio in FTS group were significantly higher than those in control group (All *P* < 0.05). However, there were no differences in the level of IgM and CD8 lymphocytes between the two groups.

**Conclusions:**

FTS improves postoperative clinical recovery and effectively inhibited release of inflammatory factors via the immune system after esophagectomy for esophageal cancer.

**Trial registration:**

ChiCTR-TRC-13003562, the date of registration: August 29, 2013.

## Background

Since its introduction in the 1990s, the concept of fast-track surgery (FTS) has gained widespread acceptance and is now considered as a standard of care. FTS also referred to as enhanced recovery after surgery (ERAS) have been implemented in order to enhance recovery, reduce morbidity and mortality rates, and shorten hospital stay after major surgery. The aim of this novel approach to perioperative patient care is to decrease the perioperative stress response to the surgical trauma and thereby leading to a decrease in complication rates in surgery. These promising clinical results lead to the question of whether the concept of FTS also results in better-preserved immune function in the postoperative course. Some researchers believe that FTS also has positive effects on the human immune system, which may result in quicker recovery of postoperative immune function [1]. Nevertheless, few clinical studies results have reported the impact of FTS on human immunity. Therefore, based on the hypothesis and present evidence of the benefits of FTS, we prospectively studied 276 patients underwent esophagectomy for esophageal cancer who either received FTS pathway or conventional pathway in the perioperative period. In addition to clinical outcome parameters, we analysed the effects of FTS on proinflammatory cytokine IL-6 and CRP levels as well as immunoglobulin and lymphocyte subgroups before surgery and on days 1, 3 and 5 after surgery.

## Methods

### Patients and procedures

This study was conducted in the Department of Thoracic Surgery at Harbin Medical University Cancer Hospital from October 2013 to December 2014. Inclusion criteria included: age ≥18 and ≤75 years, American Society of Anesthesiologists (ASA) grade I/II, body mass index (BMI) 18.5–27.5 kg/m^2^, resectable esophageal cancer (page 36, NCCN Guidelines version 1.2013). However, we found in our previous clinical study involving patients with confounding factors that such factors might have a great impact on the results, such as immunological parameters for both controlled and observational groups. Therefore, some patients needed to be excluded from our study. The exclusion criteria of the study were as follows: patients with known immunological dysfunction (advanced liver disease (decompensated cirrhosis, portal hypertension or hepatocellular carcinoma), HIV infection, hepatitis C virus infection), pulmonary insufficiency (An acute or chronic condition marked by impaired pulmonary function, characterized by elevated carbon dioxide or decreased oxygen, or both), unresectable esophageal cancer (page 36, NCCN Guidelines version 1.2013), ASA III-IV, Karnofsky index less than 60, BMI less than 18.5 kg/m^2^, and age of 65–75 years with hypertension, diabetes, or vascular disease. Two hundred and seventy-six patients who were clinically diagnosed as having esophageal cancer were assigned to two groups comprising 138 patients each: FTS group and conventional group. Enrolled patients were randomly assigned to two groups using computer-generated random numbers. For approximately equal allocation to the two treatments, we took odd and even numbers to indicate treatments A (FTS group) and B (conventional group), respectively. The patients were admitted to different peri-operative care wards based on the computer-generated random numbers when they were admitted; specifically, 138 patients were randomized to traditional protocol wards and 138 to the FTS surgery wards.

**Fig. 1:**
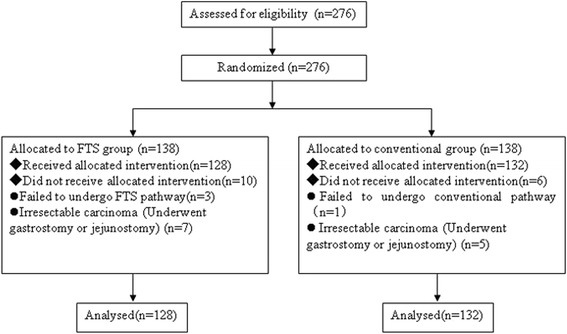
Patient flow throughout the study

Ten patients in the FTS group and 6 patients in the conventional group failed to undergo FTS and conventional pathway. Most of them did not undergo esophagectomy as expected. The final study population included 260 patients (Fig. 1). The relevant characteristics of patients and the types of surgery are listed in Table 1. Gastroscope and barium meal of the upper gastrointestinal tract were systematically performed for tumors before operations. All patients underwent further work-up to assess the medical operability. This included evaluation of pulmonary and cardiac function, cervical and abdominal ultrasonography, chest computed tomography, and hematological examinations.

**Table 1:** Characteristics of patients and their diagnosis

Characteristics	FTS group (*n* = 128)	Conventional group (*n* =132)	*P* value
Median age	56.43 ± 13.28	55.72 ± 10.34	0.252
Gender			0.973
Male	103 (80.5)	106 (80.3)	
Female	25 (19.5)	26 (19.7)	
Weight (kg)	67.53 ± 14.37	66.45 ± 13.56	0.448
BMI (kg/m^2^)	22.53 ± 2.85	22.89 ± 2.56	0.272
Operating time (min)	168.98 ± 30.62	172.33 ± 24.67	0.438
Blood loss (ml)	302.54 ± 88.48	312. 33 ± 76.73	0.727
Operative incision			0.749
One	44 (34.4)	48 (36.4)	
Two	58 (45.3)	62 (46.9)	
Three	26 (20.3)	22 (16.7)	
TNM			0.773
I	39 (30.5)	36 (27.2)	
II	71 (55.4)	79 (59.8)	
III	18 (14.1)	17 (12.8)	
IV	0 (0)	0 (0)	
Pathology			0.737
Adenocarcinoma	7 (5.5)	8 (6.1)	
Squamous cell carcinoma	116 (90.6)	121 (91.6)	
Other	5 (3.9)	3 (2.3)	
Tumor location			0.921
Upper esophagus	12 (9.4)	14 (10.6)	
Mid esophagus	73 (57.0)	76 (57.6)	
Distal esophagus	43 (33.6)	42 (31.8)	
Neoadjuvant therapy			0.988
Yes	68 (53.1)	70 (53.0)	
No	60 (46.9)	62 (47.0)	
Neoadjuvant regimen			0.923
Neoadjuvant chemoradiotherapy	27 (39.7)	25 (35.7)	
Neoadjuvant chemotherapy	41 (60.3)	45 (64.3)	
pCR after Neoadjuvant therapy	15 (22.1)	17 (24.2)	0.776
Surgical approach			0.953
Conventional thoracotomy	62 (48.4)	66 (50.0)	
Hybrid VATS	41 (32.0)	42 (31.8)	
Pure VATS	25 (19.6)	24 (18.2)	

*Variables* were expressed as the mean ± SD

*pCR* pathologic complete response rates

The FTS pathway used was developed by our cooperation team based on a previous protocol [2]. The principles of the FTS and conventional pathways are described in Table 2, and the principles of the postoperative FTS and conventional pathways are described in Table 3. The study was approved by the Research Ethics Committee of Harbin Medical University, and written informed consent was obtained from all subjects.

**Table 2:** Principles of FTS pathway and conventional pathway

	FTS pathway	Conventional pathway
Preoperative education	Patients were educated systematically by the esophageal clinical nurse consultant;	Patients were educated in the standard manner
Day before surgery		
Diet	Last drink 2 h and diet 6 h before operation	Last drink and diet at midnight
Fructose and protein loading	Yes	No
Day of surgery		
Nasogastric tube	No routine use of nasogastric tube	Routine use of nasogastric tube
Pre-anesthetic medication	No	Diazepam 10 mg
Anesthesia	General anesthesia + Epidural anesthesia;	General anesthesia;
Maintaining normothermia	Yes	No
Transfusion	Autologous blood transfusion or limit allogenic blood transfusion	Allogenic blood transfusion
Abdomen tube	No routine use of drains	Routine placement; Remove at POD3
Cervical tube	No routine use of drains	Routine placement; Remove at POD2
Early postoperative care	Patient sent to floor	Patient sent to ICU
Analgesia	Epidural PCA	Analgesia by morphine or vein PCA
Enteral nutrition	Jejunostomy tube feeding	Nasojejunal tube feeding

**Table 3:** Daily guideline of postoperative care of patients with FTS pathway vs conventional pathway

Day	FTS pathway	Conventional pathway
POD1	Jejunostomy tube feeding 500 mL (starting at 20 mL/h) Early postoperative mobilization program (>2 h out of bed) Physical therapy and nebulizers Remove urine catheter Head of bed put at 30° Supply albumin Chest tube to suction Promoted to lung recruitment	Total parenteral nutrition Bed rest Gastrointestinal decompression Closed thoracic drainage
POD2	Jejunostomy tube feeding 1000 mL (40 mL/h) Chest tube to suction Expand mobilization (>4 h out of bed) Continue physical therapy and nebulizers Continue supply albumin	Nasojejunal tube feeding 500 mL (starting at 20 mL/h) Remove urine catheter With help, sit in the chair 2 times during the day for at least 30 min each time Gastrointestinal decompression Closed thoracic drainage
POD3	Jejunostomy tube feeding 1500 mL (60–80 mL/h) Remove chest tube Remove epidural catheter Expand mobilization (>6 h out of bed) Continue physical therapy and nebulizers Continue supply albumin	Nasojejunal tube feeding 1000 mL (40 mL/h) Sit in the chair 3 times for at least 30–60 min each time. With help, walk twice in the hallway. Do deep breathing exercise Remove nasogastric tube Closed thoracic drainage
POD4	Gastrograffin opacification of upper gastrointestine If swallow shows no leak, advance patient to oral drink Jejunostomy tube feeding 1500 mL (60–80 mL/h) Continue physical therapy and nebulizers Education on aspiration precaution Education on chewing and swallowing	Nasojejunal tube feeding 1000 mL (40 mL/h) Sit in the chair 3 times today for at least 30–60 min each time. Walk the length of the hallway 3 times Continue to do breathing exercises Closed thoracic drainage
POD5	Jejunostomy tube feeding 1500 mL (60–80 mL/h) Advance patient to a full liquid diet Continue aspiration precautions Continue physical therapy and nebulizers	Nasojejunal tube feeding 1500 mL (60–80 mL/h) Walk the length of the hallway 4–5 times. Sit in the chair 3 times today for at least 30–60 min Continue to do breathing exercises
POD6	Increase liquid diet Decrease jejunostomy tube feeding (500 ml or 1000 ml) Continue aspiration precautions Continue physical therapy and nebulizers	Nasojejunal tube feeding 1500 mL (60–80 mL/h) Remove chest tube Walk the length of the hallway 4–5 times. Sit in the chair 3 times today for at least 30–60 min Continue to do breathing exercises
POD7	Remove jejunostomy tube Full liquid diet Discharge home on soft diet and liquid diet Continue aspiration precautions	Gastrograffin opacification of upper gastrointestine If swallow shows no leak, advance patient to oral drink Nasojejunal tube feeding 1500 mL (60–80 mL/h) Expand mobilization (>4 h out of bed) Continue to do breathing exercises
POD8		Increase liquid diet Decrease jejunostomy tube feeding (500 ml or 1000 ml) Expand mobilization (>6 h out of bed) Continue to do breathing exercises
POD9		Remove nasojejunal tube Full liquid diet Expand mobilization (>6 h out of bed) Continue to do breathing exercises
POD10-11		Soft diet and liquid diet Nearly out of bed Observe whether there is delayed anastomotic leakage
POD12		Discharge home on soft diet and liquid diet

### Clinical parameters

The post-operative hospital stay defined as time spent in the hospital from the day of operation to the day of hospital discharge, including readmission stay within 30 days postoperatively. The complications were defined as atrial arrhythmia, anastomotic leak, ileus, pneumonia, ARDS and incision infection. Readmission rate was also recorded. Pain while coughing, staying in bed or during exercise was judged by the patients three times daily until day 5 after surgery using the numeric rating scale (0, no pain to 10, maximum pain). The perioperative hospital charges included surgery, anesthesia, drugs, auxiliary examination (including laboratory and radiology), and care costs, but didn’t include neoadjuvant therapy costs.

### Protocol for esophageal cancer

The diagnostic and therapeutic protocols for patients with esophageal cancer at the authors’ institution is based on NCCN Guidelines version 1.2013 (page 36–37). Since the R0-resection rate and long-term outcome of patients with T3/T4 tumors is poor with primary resection, multimodal therapeutic concepts with preoperative chemotherapy or combined radiochemotherapy or both are employed in these patients.

### Pro-inflammatory parameters

Peripheral venous blood samples were collected in serum collection tubes (Kabe) and were subsequently centrifuged at 300 × g for 15 min at 4 °C and serum samples were subsequently stored at −80 °C until assayed for IL-6.

Circulating serum IL-6 levels were determined using sandwich enzyme-linked immunosorbent assay (Biosource, Nivelles, Belgium) as described by the manufacturer. CRP was measured with the immunoturbidimetric method (Olympus, Hamburg, Germany).

### Immunological parameters

Blood samples were taken on the day before surgery as well as on days 1, 3 and 5 after surgery. All blood samples were taken from peripheral veins at 6 a.m., before breakfast. The humoral immunologic factors tested in our study included serum globulin, immunoglobulin G (IgG), immunoglobulin M (IgM), immunoglobulin A (IgA).

### Lymphocyte subpopulation parameters

Lymphocyte subpopulations (CD3, CD4, and CD8 lymphocytes, and the CD4/CD8 ratio) were determined by flow cytometry (Becton Dickinson, San Jose, CA, USA). The monoclonal antibodies used for immunophenotyping were purchased from Becton Dickinson and conjugated to the fluorochromes, fluorescein isothiocyanate or phycoerythrin. The fluorescence was measured using a FACScalibur (Becton Dickinson) within 60 min of processing of the samples. Fluorescent-activated cell sorting analysis was carried out on a FACScalibur flow cytometer. A minimum of 10,000 cells were measured for each determination.

### Discharge and follow-up

Patients were discharged only if they could tolerate a semiliquid or soft diet and walk freely in the ward. Data were collected prospectively and retrieved from our database. Complete follow-up was available until 1 month after surgery.

### Statistical analysis

Outcome data were analyzed with the use of repeat measurement ANOVA for continuous variables and chi-square test or Fisher’s exact test for categorical variables. All analyses were performed with the statistical package SPSS (version 13.0; Chicago, IL). A *P* value of <0.05 was considered significant.

## Results

In all, 260 patients finished the study, including 128 patients in the FTS group and 132 patients in the conventional group. Ten patients were excluded from the FTS group and six patients from the conventional group (Fig. 1). No significant differences were observed in sex, age, weight, BMI, operating time, blood loss, operative incision, tumor TNM stage, tumor pathology, tumor location and neoadjuvant therapy between the two groups (Table 1).

### Clinical parameters

Postoperative hospital stay in patients randomized to the FTS group was significantly shorter than in the conventional group (*P* < 0.05). The mean charge for perioperative hospital stay was 35823.62 ± 3598.81 renminbi (RMB) for the FTS group, which was significantly less than the cost of 41032.73 ± 4013.32 RMB for the conventional group (*P* <0.05). Incision pain according to the Numeric Rating Scale was lower in patients of the FTS group than in those of the conventional group (*P* <0.05). And we had compared the degree of pain among three different surgical approaches: pure video-assisted thoracic surgery (VATS), hybrid VATS, and conventional thoracotomy. In the early postoperative period, pure VATS was shown to be the least painful of the three surgical approaches. No statistically significant differences were detected in postoperative complications between the two groups (*P* < 0.05). There was, however, a trend toward more postoperative complications (9.8 %) in patients undergoing conventional pathway (Table 4). According to Clavien-Dindo classification, 11 (8.6 %) patients in FTS group and 16 (12.1 %) patients in conventional group suffered stageI, II and III complications respectively. Two patient in FTS group and three patient in the conventional group was diagnosed atrial arrhythmia by an ECG recording (I). Two patient in FTS group and two patient in the conventional group showed incision infection (I). One patient in the conventional group developed postoperative paralytic ileus and required reinsertion of a nasogastric tube, and this was resolved by restricting intake and parenteral nutrition (II). Three patients in FTS group and four patients in the conventional group had pneumonia and this was resolved by physical therapy and antibiotic treatment (II). Anastomotic leak occurred in two patient in FTS group and three patients in the conventional group, and this was resolved by endoscopic treatment (III). Two patients in FTS group and three patients in the conventional group had ARDS and were treated with mechanical ventilation in the Intensive Care Unit (IV).

**Table 4:** Comparison of outcome of two group

Outcomes	FTS group (*n* = 128)	Conventional group (*n* =132)	*P* value
Postoperative hospital stay (d)	7.62 ± 1.38	12.56 ± 1.92	0.000
Hospitalization expenditure (RMB)	35823.62 ± 3598.81	41032.73 ± 4013.32	0.000
Incision pain scale (NRS)	4.72 ± 1.94	7.66 ± 1.59	0.000
Morbidity	11 (8.6)	16 (12.1)	0.351
Atrial arrhythmia	2	3	
Ileus	0	1	
Pneumonia	3	4	
Anastomotic leak	2	3	
Incision infection	2	2	
ARDS	2	3	
Mortality	2 (1.6)	2 (1.5)	1.000
30-day readmission rate	3 (2.3)	3 (2.3)	1.000

*Variables* were expressed as the mean ± SD

The Numeric Rating Scale (NRS) is an 11–point (0–10) scale for patient self-reporting of pain. It is for adults and children 10 years old or older

*RMB* Ren Min Bi or China Yuan

### Pro-inflammatory parameters

On PODs 1 and 3, statistically significant differences were found in levels of IL-6 and CRP with the FTS group having lower levels than in the conventional group (*P* <0.05). On POD 7, the level of CRP was lower in the FTS group than that in the conventional group (*P* <0.05) (Table 5).

**Table 5:** Comparison of inflammatory markers in two groups

Factor and time	FTS group (*n* = 128)	Conventional group (*n* = 132)	*P* value
IL-6 (ng/L)			
Before surgery	53.83 ± 21.66	55.73 ± 20.37	0.585
POD1	121.74 ± 22.57	138.77 ± 21.53*	0.000
POD3	142.37 ± 25.09	154.90 ± 24.33*	0.035
POD7	116.70 ± 22.39	122.79 ± 25.64	0.412
CRP (μg/L)			
Before surgery	4.97 ± 1.33	4.85 ± 1.43	1.000
POD1	65.57 ± 13.37	74.61 ± 14.71*	0.034
POD3	136.79 ± 23.34	155.38 ± 28.75*	0.012
POD7	51.83 ± 17.66	62.36 ± 18.37*	0.042

*Variables* were expressed as the mean ± SD

* *P* <0.05

### Immunological parameters

There were no significant differences in the post-operativelevel and pre-operative level of IgM and CD8 between the two groups. On PODs 1 and 3, statistically significant differences were found in the levels of IgG, IgA, CD3, CD4 and CD4/CD8 ratio with the FTS group having higher levels than the conventional group (all *P* <0.05). On POD 3, the level of serum globulin was higher in the FTS group than that in the conventional group (*P* <0.05) (Table 6). We performed subgroup analysis based on neoadjuvant or not, as well as MIE (Minimally invasive esophagectomy) or not for avoiding bias, and we came to similar conclusions after data analysis (Tables 7, 8, 9 and 10).

**Table 6:** Comparison of immunologic factors in two groups

Factor and time	FTS group (*n* = 128)	Conventional group (*n* = 132)	*P* value
Globulin (g/l)			
Before surgery	28.37 ± 3.93	27.32 ± 4.33	0.943
POD1	22.74 ± 3.93	22.39 ± 3.82	1.000
POD3	25.66 ± 2.94	22.21 ± 2.99*	0.038
POD7	29.95 ± 3.85	27.97 ± 4.41	0.537
IgG (g/l)			
Before surgery	14.38 ± 2.78	15.33 ± 3.79	0.573
POD1	8.97 ± 1.79	6.11 ± 1.38*	0.033
POD3	11.02 ± 3.53	8.17 ± 2.94*	0.002
POD7	14.53 ± 3.81	13.02 ± 3.73	0.741
IgA (g/l)			
Before surgery	2.79 ± 0.52	2.98 ± 0.35	0.757
POD1	1.81 ± 0.43	1.65 ± 0.17*	0.012
POD3	2.08 ± 0.54	1.76 ± 0.47*	0.003
POD7	2.58 ± 0.47	2.62 ± 0.39	0.637
IgM (g/l)			
Before surgery	1.21 ± 0.35	1.24 ± 0.44	1.000
POD1	0.82 ± 0.39	0.89 ± 0.43	0.964
POD3	0.75 ± 0.22	0.77 ± 0.31	0.543
POD7	1.18 ± 0.59	1.23 ± 0.48	0.424
CD3			
Before surgery	55.99 ± 2.72	57.83 ± 2.64	0.813
POD1	49.92 ± 2.75	46.01 ± 2.83*	0.042
POD3	51.83 ± 2.42	48.02 ± 2.51*	0.019
POD7	55.05 ± 2.69	53.83 ± 2.71	0.737
CD4			
Before surgery	45.58 ± 3.92	44.97 ± 4.32	0.958
POD1	33.26 ± 4.72	30.37 ± 5.21*	0.039
POD3	39.39 ± 4.81	34.34 ± 5.72*	0.012
POD7	43.76 ± 4.38	42.87 ± 3.98	0.887
CD8			
Before surgery	26.73 ± 4.85	26.08 ± 3.97	1.000
POD1	23.72 ± 4.33	24.42 ± 4.74	0.958
POD3	23.76 ± 3.83	22.73 ± 4.65	0.832
POD7	28.73 ± 4.38	27.62 ± 3.83	0.732
CD4/CD8			
Before surgery	1.53 ± 0.33	1.48 ± 0.42	0.739
POD1	1.45 ± 0.31	1.22 ± 0.45*	0.042
POD3	1.52 ± 0.45	1.39 ± 0.30*	0.023
POD7	1.58 ± 0.32	1.53 ± 0.54	0.865

*Variables* were expressed as the mean ± SD

* *P* <0.05

**Table 7:** Comparison of inflammatory markers and immunologic factors in two groups without neoadjuvant

Factor and time	FTS group (*n* = 60)	Conventional group (*n* = 62)	*P* value
IL-6 (ng/L)			
Before surgery	52.13 ± 25.54	54.67 ± 22.36	0.651
POD1	123.43 ± 20.73	139.26 ± 22.92*	0.007
POD3	144.05 ± 26.39	156.25 ± 25.38*	0.032
POD7	117.23 ± 21.29	123.36 ± 25.56	0.321
CRP (mg/L)			
Before surgery	4.92 ± 1.53	4.83 ± 1.72	0.953
POD1	66.37 ± 12.93	75.52 ± 14.88*	0.023
POD3	138.84 ± 22.04	156.58 ± 29.21*	0.019
POD7	53.84 ± 16.95	64.06 ± 16.74*	0.031
Globulin (g/l)			
Before surgery	28.82 ± 3.93	27.97 ± 5.21	0.643
POD1	22.25 ± 3.83	21.09 ± 3.90	0.231
POD3	25.72 ± 3.08	22.68 ± 2.89*	0.026
POD7	28.56 ± 2.25	27.74 ± 4.04	0.447
IgG (g/l)			
Before surgery	15.82 ± 2.98	14.18 ± 3.69	0.585
POD1	8.87 ± 1.65	6.32 ± 1.19*	0.025
POD3	10.88 ± 3.37	7.94 ± 2.87*	0.000
POD7	14.51 ± 2.83	13.97 ± 3.63	0.231
IgA (g/l)			
Before surgery	2.76 ± 0.33	2.85 ± 0.27	0.654
POD1	1.71 ± 0.54	1.45 ± 0.14*	0.013
POD3	1.98 ± 0.47	1.67 ± 0.52*	0.002
POD7	2.53 ± 0.33	2.42 ± 0.38	0.585
IgM (g/l)			
Before surgery	1.24 ± 0.33	1.19 ± 0.46	0.841
POD1	0.85 ± 0.49	0.81 ± 0.32	0.432
POD3	0.77 ± 0.17	0.71 ± 0.26	0.233
POD7	1.15 ± 0.53	1.21 ± 0.50	0.190
CD3+			
Before surgery	57.92 ± 2.63	58.23 ± 2.48	0.764
POD1	48.87 ± 2.51	46.13 ± 2.78*	0.047
POD3	51.17 ± 2.20	48.19 ± 2.31*	0.013
POD7	55.59 ± 2.28	53.07 ± 2.84	0.230
CD4+			
Before surgery	44.28 ± 3.48	44.73 ± 4.52	0.864
POD1	34.95 ± 4.72	31.07 ± 5.09*	0.020
POD3	38.52 ± 3.61	34.54 ± 5.22*	0.016
POD7	43.38 ± 5.86	42.48 ± 3.84	0.382
CD4+/CD8+			
Before surgery	1.64 ± 0.32	1.55 ± 0.49	0.872
POD1	1.43 ± 0.21	1.20 ± 0.51*	0.036
POD3	1.54 ± 0.55	1.41 ± 0.34*	0.018
POD7	1.57 ± 0.24	1.51 ± 0.28	0.341

*Variables* were expressed as the median ± quartile

* *P* <0.05

**Table 8:** Comparison of inflammatory markers and immunologic factors in two groups with neoadjuvant

Factor and time	FTS group (*n* = 68)	Conventional group (*n* = 70)	*P* value
IL-6 (ng/L)			
Before surgery	50.57 ± 21.45	51.42 ± 19.83	0.979
POD1	119.54 ± 18.62	131.42 ± 21.58*	0.004
POD3	138.42 ± 22.73	150.48 ± 21.97*	0.043
POD7	112.52 ± 19.43	116.43 ± 22.57	0.573
CRP (mg/L)			
Before surgery	4.53 ± 1.24	4.45 ± 1.29	0.942
POD1	61.32 ± 13.77	70.52 ± 11.56*	0.032
POD3	132.73 ± 21.56	150.49 ± 25.72*	0.013
POD7	49.78 ± 15.84	60.43 ± 16.38*	0.035
Globulin (g/l)			
Before surgery	25.67 ± 3.34	25.82 ± 4.39	1.000
POD1	21.43 ± 3.63	20.21 ± 3.45	0.760
POD3	24.76 ± 3.82	21.64 ± 2.94*	0.023
POD7	25.73 ± 3.35	25.87 ± 4.09	0.848
IgG (g/l)			
Before surgery	13.76 ± 3.13	13.62 ± 3.42	0.858
POD1	7.42 ± 1.42	5.23 ± 1.35*	0.031
POD3	9.42 ± 3.53	6.44 ± 2.95*	0.001
POD7	12.56 ± 2.67	11.94 ± 3.83	0.656
IgA (g/l)			
Before surgery	2.19 ± 0.29	2.21 ± 0.25	0.769
POD1	1.61 ± 0.37	1.34 ± 0.25*	0.017
POD3	1.85 ± 0.42	1.51 ± 0.51*	0.004
POD7	2.04 ± 0.37	2.15 ± 0.42	0.753
IgM (g/l)			
Before surgery	1.14 ± 0.31	1.11 ± 0.41	0.893
POD1	0.71 ± 0.39	0.72 ± 0.33	0.957
POD3	0.67 ± 0.22	0.64 ± 0.32	0.873
POD7	1.09 ± 0.43	1.10 ± 0.42	0.673
CD3+			
Before surgery	55.62 ± 2.43	56.82 ± 2.58	0.875
POD1	46.73 ± 2.52	44.09 ± 2.03*	0.043
POD3	49.82 ± 2.43	46.17 ± 2.35*	0.033
POD7	53.85 ± 2.05	54.04 ± 2.73	0.482
CD4+			
Before surgery	42.43 ± 3.57	42.48 ± 4.92	0.803
POD1	32.52 ± 4.13	29.12 ± 5.02*	0.025
POD3	35.32 ± 3.90	32.55 ± 5.02*	0.013
POD7	41.42 ± 5.24	40.98 ± 4.27	0.484
CD4+/CD8+			
Before surgery	1.52 ± 0.41	1.55 ± 0.45	0.842
POD1	1.33 ± 0.36	1.11 ± 0.41*	0.027
POD3	1.47 ± 0.56	1.32 ± 0.37*	0.025
POD7	1.50 ± 0.36	1.52 ± 0.29	0.562

*Variables* were expressed as the median ± quartile

* *P* <0.05

**Table 9:** Comparison of inflammatory markers and immunologic factors in two groups without MIE

Factor and time	FTS group (*n* = 63)	Conventional group (*n* = 65)	*P* value
IL-6 (ng/L)			
Before surgery	53.26 ± 24.18	53.58 ± 23.14	0.842
POD1	123.73 ± 22.69	139.42 ± 24.78*	0.006
POD3	143.31 ± 25.42	155.79 ± 24.42*	0.043
POD7	117.73 ± 22.45	123.57 ± 23.84	0.571
CRP (mg/L)			
Before surgery	4.94 ± 1.48	4.86 ± 1.32	0.943
POD1	66.42 ± 13.73	75.75 ± 13.57*	0.032
POD3	137.76 ± 24.73	156.79 ± 27.42*	0.015
POD7	52.75 ± 15.79	63.73 ± 15.2*	0.042
Globulin (g/l)			
Before surgery	27.82 ± 3.59	28.71 ± 5.33	0.875
POD1	21.63 ± 3.54	21.79 ± 3.82	0.941
POD3	25.59 ± 3.14	22.79 ± 2.98*	0.043
POD7	28.73 ± 3.08	27.95 ± 4.24	0.545
IgG (g/l)			
Before surgery	14.59 ± 3.28	13.97 ± 3.82	0.833
POD1	8.93 ± 1.59	6.47 ± 1.25*	0.034
POD3	10.51 ± 3.56	7.82 ± 2.96*	0.000
POD7	13.75 ± 2.97	13.66 ± 3.32	0.741
IgA (g/l)			
Before surgery	2.65 ± 0.41	2.75 ± 0.32	1.000
POD1	1.78 ± 0.49	1.41 ± 0.25*	0.009
POD3	1.94 ± 0.46	1.65 ± 0.43*	0.004
POD7	2.43 ± 0.35	2.49 ± 0.41	0.842
IgM (g/l)			
Before surgery	1.26 ± 0.41	1.21 ± 0.33	0.892
POD1	0.83 ± 0.38	0.85 ± 0.36	0.731
POD3	0.72 ± 0.19	0.71 ± 0.22	1.000
POD7	1.19 ± 0.49	1.24 ± 0.42	0.766
CD3+			
Before surgery	58.03 ± 2.52	57.43 ± 2.67	0.852
POD1	48.93 ± 2.47	45.89 ± 2.49*	0.048
POD3	51.67 ± 2.33	47.87 ± 2.52*	0.011
POD7	54.68 ± 2.32	53.25 ± 2.93	0.473
CD4+			
Before surgery	44.37 ± 3.52	44.52 ± 4.31	0.768
POD1	36.43 ± 4.28	32.66 ± 5.14*	0.028
POD3	39.13 ± 4.52	33.95 ± 5.38*	0.004
POD7	42.57 ± 4.38	42.64 ± 3.95	1.000
CD4+/CD8+			
Before surgery	1.59 ± 0.41	1.54 ± 0.43	0.892
POD1	1.47 ± 0.23	1.22 ± 0.41*	0.032
POD3	1.51 ± 0.45	1.40 ± 0.51*	0.012
POD7	1.54 ± 0.29	1.57 ± 0.21	0.842

*Variables* were expressed as the median ± quartile

* *P* <0.05

**Table 10:** Comparison of inflammatory markers and immunologic factors in two groups with MIE

Factor and time	FTS group (*n* = 65)	Conventional group (*n* = 67)	*P* value
IL-6 (ng/L)			
Before surgery	52.33 ± 24.37	53.42 ± 22.73	0.764
POD1	119.34 ± 21.57	134.42 ± 22.53*	0.005
POD3	140.05 ± 25.63	151.67 ± 24.52*	0.035
POD7	113.72 ± 22.42	117.66 ± 25.74	0.152
CRP (mg/L)			
Before surgery	4.62 ± 1.63	4.53 ± 1.57	0.842
POD1	62.42 ± 13.83	71.42 ± 14.32*	0.027
POD3	134.73 ± 22.57	153.82 ± 25.62*	0.017
POD7	50.46 ± 15.73	61.43 ± 15.66*	0.012
Globulin (g/l)			
Before surgery	27.72 ± 3.55	27.58 ± 5.14	0.942
POD1	23.57 ± 3.73	22.64 ± 3.73	0.543
POD3	25.42 ± 3.95	23.31 ± 3.52*	0.021
POD7	28.04 ± 2.76	27.87 ± 4.13	0.735
IgG (g/l)			
Before surgery	14.63 ± 2.57	14.03 ± 3.1	0.872
POD1	9.43 ± 1.25	7.62 ± 1.17*	0.028
POD3	11.82 ± 3.52	8.73 ± 2.94*	0.002
POD7	14.42 ± 2.76	13.52 ± 3.33	0.542
IgA (g/l)			
Before surgery	2.57 ± 0.31	2.77 ± 0.29	0.767
POD1	1.83 ± 0.41	1.54 ± 0.22*	0.015
POD3	1.99 ± 0.35	1.69 ± 0.42*	0.000
POD7	2.55 ± 0.32	2.52 ± 0.32	0.652
IgM (g/l)			
Before surgery	1.28 ± 0.31	1.21 ± 0.36	0.853
POD1	0.94 ± 0.43	0.92 ± 0.35	0.482
POD3	0.87 ± 0.21	0.81 ± 0.32	0.353
POD7	1.25 ± 0.46	1.22 ± 0.52	0.320
CD3+			
Before surgery	57.43 ± 2.82	58.12 ± 2.73	0.873
POD1	49.69 ± 2.43	47.02 ± 2.57*	0.044
POD3	53.38 ± 2.57	49.93 ± 2.65*	0.017
POD7	56.73 ± 2.52	56.48 ± 2.49	1.000
CD4+			
Before surgery	44.52 ± 3.46	44.37 ± 4.41	1.000
POD1	35.87 ± 4.15	32.35 ± 4.89*	0.022
POD3	39.98 ± 3.73	35.43 ± 4.64*	0.013
POD7	44.73 ± 5.02	43.76 ± 3.92	0.859
CD4+/CD8+			
Before surgery	1.58 ± 0.30	1.52 ± 0.42	0.973
POD1	1.49 ± 0.24	1.26 ± 0.43*	0.031
POD3	1.64 ± 0.45	1.45 ± 0.28*	0.015
POD7	1.56 ± 0.32	1.50 ± 0.37	0.458

*Variables* were expressed as the median ± quartile

*P <0.05

## Discussion

Recent clinical data indicate that FTS leads to shorter postoperative length of hospital stay, faster recovery of gastrointestinal function as well as reduced morbidity and mortality rates [1, 3, 4]. To date, no study has focused on the effects of FTS on immune function after esophagectomy for esophageal cancer. This study was initiated to determine whether FTS results in improved clinical and immunological outcome of patients undergoing esophagectomy for esophageal cancer.

The normal inflammatory response, more commonly known as the stress response, was first described by Sir David Cuthbertson [5]. Despite variability in the intensity of the stress response, the timeline of events remains remarkably similar, with few exceptions. Virtually all mediators of inflammation and metabolism peak about postinjury day 2 and then return to baseline levels by postinjury days 6–7. The inflammatory response may obviously be important for wound healing and resistance to infection, but on the other hand may have undesirable effects by enhancing pain and leading to fatigue and sleep disturbances [6]. Inflammation is triggered when innate immune cells detect infection or tissue injury. We found most of immune markers decreased under condition of the increase of pro-inflammatory cytokines. This research into the mechanisms that immune impact may provide a novel therapeutic pathway that can alter the nature and time course of the stress response.

Perioperative intervention improvements that might be contributed to the immunologic protection, including taking a carbohydrate rich drink before surgery, early enteral nutrition and epidural analgesia. Before surgery, patients often already have poor nutritional status and low immune function. For patients with esophageal cancer, their nutritional status will be worsened after surgeries, along with the decrease of the cellular and humoral immunity [7]. Some studies indicated that taking a carbohydraterich drink before surgery could reduce the endocrine catabolic response and improve insulin resistance, improving surgical results and hastening recovery [8, 9]. Gut is regarded as a central organ after surgical stress; also, among the intestinal mucosal barrier functions, immune barrier plays an important role. the functions of small intestine often return normal 6–12 h after the surgery, which supports the early application of enteral nutrition (EN) after surgery [10]. Clinically, EN is applied to facilitate the improvement of nutritional status, restoration of immune function, and protection of intestinal mucosal barrier after the surgeries. A recent study verified that partial EN support during perioperation will not only improve the postoperative nutritional status and immune function, but also moderate the inflammatory response of gastric cancer patients after operative trauma [11]. Beier-Holgersen et al. [12] proved early postoperative enteral nutrition had an important influence on immediate unspecific cellular immunity and had an activating effect on specific cellular immunity. In our current randomized and controlled study, we provided EN with postoperative patients in the FTS group on POD1 and in the conventional group on POD2. The immune globulins (IgA and IgG) and T lymphocyte subsets (CD3, CD4 and CD4/CD8) at POD1, 3 were higher in the FTS group than them in the conventional group, showing a fast recovery of immunity. We hypothesized that the fast recovery of immunity might be ascribed to the fact that the earlier activation of the immune system by earlier EN after surgery.

It has been verified that nociception and proinflammatory cytokines play a mutual up-regulatory role [13]. Therefore, pain management may influence the immune response in the postoperative period. It has been reported that the alterations in lymphocyte subsets and the increase in white cell counts induced by surgery and general anesthesia can be prevented by epidural analgesia [14, 15]. Furthermore, Beilin et al. [16] reported that patients treated with patient-controlled analgesia (PCA) exhibited attenuated proinflammatory cytokine response in the postoperative period. Also Khaled et al. [17] showed that thoracic epidural analgesia reduced the systemic pro-inflammatory response and provided optimal post-operative pain relief. In major surgery, however, the effect of epidural anesthesia and analgesia on attenuation of the stress response and preservation of immune function is controversial.

The difference in immune function between the two groups was also statistically significant. There are several factors that may account for these differences. The duration of surgery and anesthesia, extent of tissue injury, and blood loss are usually great in patients undergoing esophagectomy. Blood transfusion is another factor in inducing immunosuppression. There is increasing evidence to suggest that perioperative blood transfusion may have an immunomodulatory effect. A previous study [18] of patients undergoing esophageal resection for carcinoma has demonstrated a significantly worse prognosis for those receiving a blood transfusion independent of disease stage or the presence of major complications. It has been suggested that the immunosuppression induced by transfusion results from both an early unspecific immunosuppression mediated by monocytes and a later phase induced from increased suppressor T cell activity. Blood transfusion has been shown to lower the CD4 to CD8 ratio [19]. In addition, prostaglandin E2 levels are increased after transfusion [20]. This may result in a direct inhibition of interleukin-2 production from CD4 cells with subsequent effect, as interleukin-2 is obligatory for natural killer cell activity. In addition, it shows that transfusion of more than 3 units of blood can adversely affect survival [21]. Therefore, every effort should be made to limit the amount of blood transfused to the minimum requirement.

Cellular immunity is mediated by lymphocytes and transferred by the cells of immunized people. Humoral immunity is mediated by antibodies and transferred by the sera of immunized people. Impairment of cellular and humoral immune system may lead to infections secondary to surgery, progression of malign tumors, and emergence of opportunistic infections. IL-6 is known to be a major mediator of the acute-phase response and plasma levels of IL-6 are reportedly related to the severity of surgical trauma [22]. It can stimulate the liver to synthesize C-reactive protein (CRP), enhance inflammatory reaction by promoting B cell differentiation and antibody formation, and assist the T cells to produce the expressions of IL-2 and its receptor [23]. In our current study, no significant difference was found in both groups of immunological parameters before surgery and POD7. At PODs 1 and 3, the immune function indicators decreased in both groups. In the FTS group, the immune globulins (IgA and IgG) and T lymphocyte subsets (CD3, CD4 and CD4/CD8) were significantly higher than those in the conventional group (*P* < 0.05), whereas IL-6 and CRP were significantly lower (*P* < 0.05). Therefore, the FTS is helpful to improve the immune function in patients undergoing esophagectomy for esophageal cancer.

Another powerful technique to reduce inflammatory responses is decreasing the wound size by minimal invasive surgery. It is well established that this may reduce pain and inflammatory responses especially proinflammatory cytokines (IL-6 etc.) [24]. Inflammatory responses contribute to pain, fatigue and organ dysfunction. Minimally invasive surgery also reduces the inflammatory response, although the contribution that this plays in the context of fast-track programmes in esophageal surgery is difficult to interpret. We found that the immune function of patients is mildly affected by minimally invasive surgery. No significant differences were observed in immune function between the minimally invasive surgery group and the open surgery group. Of interest, a recent randomized blinded study by Basse et al. showed that FTS leads to similar clinical and functional results in patients undergoing open as well as laparoscopic colorectal resections [25]. This indicates that the clinical advantages of laparoscopic surgery may be of only minor importance for postoperative recovery if the concept of FTS surgery is used for patients after major surgery. Minimal invasive surgery on a procedure-specific basis therefore represent a major opportunity to further enhance recovery and reduce morbidity in the future, especially when combined with other aspects of the fast-track methodology.

Our results did not detect statistically significant changes in serum IgM and CD8 lymphocytes between the two groups. However, this does not mean that FTS has no effects on these factors, which may be explained by the time that blood samples were taken or the relatively small number of cases in our trial. The above factors may be differences between the two groups if we increase the time that blood samples were taken, or increase the amount of cases.

Only a small proportion of patients in our study underwent Pure VATS surgery. We have not enough evidence to demonstrate the effect of minimally invasive surgery on immunity. Therefore, the changes of immune indexes by laparoscopic and thoracoscopic surgery needs to be investigated in further studies.

## Conclusions

The beneficial clinical effects of FTS reported here support the findings of other groups and our data on perioperative immunity suggest that better-preserved cellular and humoral immunity may contribute to the improvement of postoperative results in fast-track patients. The precise mechanism of changes in immunological parameters in FTS needs further study.

## Abbreviations

ASA, American Society of Anesthesiologists; BMI, body mass index; CRP, C-reactive protein; EN, enteral nutrition; ERAS, enhanced recovery after surgery; FTS, fast-track surgery; IgA, immunoglobulin A; IgG, immunoglobulin G; IgM, immunoglobulin M; IL-6, interleukin-6; PCA, patient-controlled analgesia; POD, postoperative days
